# ADHD symptoms across adolescence: the role of the family and school climate and the *DRD4* and *5*-*HTTLPR* genotype

**DOI:** 10.1007/s00787-019-01424-3

**Published:** 2019-10-18

**Authors:** Djûke M. Brinksma, Andrea Dietrich, Annelies de Bildt, Jan K. Buitelaar, Barbara J. van den Hoofdakker, Pieter J. Hoekstra, Catharina A. Hartman

**Affiliations:** 1 Department of Child and Adolescent Psychiatry, University Medical Center Groningen, University of Groningen, Hanzeplein 1, XA10, 9713 GZ Groningen, The Netherlands; 2 Accare Child and Adolescent Psychiatry, Groningen, The Netherlands; 3 Department of Cognitive Neuroscience, Donders Institute for Brain, Cognition and Behavior, Radboud University Nijmegen Medical Center, Nijmegen, The Netherlands; 4 Karakter Child and Adolescent Psychiatry University Centre, Nijmegen, The Netherlands; 5 Department of Clinical Psychology and Experimental Psychopathology, University of Groningen, Groningen, The Netherlands; 6 Department of Psychiatry, Interdisciplinary Center Psychopathology and Emotion regulation (ICPE), University Medical Center Groningen, University of Groningen, Groningen, The Netherlands

**Keywords:** ADHD, Adolescence, Gene–environment interaction, Longitudinal study, Risk and promotive factors

## Abstract

We examined bidirectional relations between attention-deficit/hyperactivity disorder (ADHD) symptoms and family and school climate, and the possible role of *DRD4* and/or *5-HTTLPR* genotypes herein. Three-wave longitudinal data of 1860 adolescents (mean ages 11, 13.5, and 16 years) from the general population and clinic-referred cohort of TRacking Adolescents’ Individual Lives Survey were used. Using a multigroup Random Intercept Cross-Lagged Panel Model, we tested between-person (i.e., stable trait levels) and within-person (i.e., causal processes) associations across ADHD symptoms, family and school climate, and the extent to which these depended on genotype. Findings indicated no influence of genotype. Results did show significant between-person differences (ADHD symptoms with family climate *r* = .38; and school climate *r* = .23, *p* values < .001), indicating that higher stable levels of ADHD symptoms were associated with a less favorable family and school climate. Regarding within-person causal processes, ADHD symptoms predicted a less favorable family climate in early adolescence (*β *= .16, *p *< .01), while ADHD symptoms predicted a more favorable family climate in the later phase of adolescence (*β* = − .11, *p* < .01), a finding which we explain by normative developmental changes during adolescence. Overall, this study showed that negative associations between ADHD symptoms and both family and school climate are largely explained by stable between-person differences. We recommend applying the Random Intercept Cross-Lagged Path Model to developmental data to tease stable associations and change processes apart.

**Electronic supplementary material:**

The online version of this article (10.1007/s00787-019-01424-3) contains supplementary material, which is available to authorized users.

## Introduction

Attention-deficit/hyperactivity disorder (ADHD) is one of the most commonly diagnosed disorders of childhood [1]. Although on average ADHD symptoms decline after childhood, the course of symptoms differs between individuals (e.g., [2–4]). The course of ADHD symptoms is explained by genetic and environmental influences [5, 6], including family functioning (e.g., [7, 8]). It is well-documented, although based on predominantly cross-sectional studies, that ADHD symptoms and adverse family circumstances may co-occur (e.g., [9–13]). Longitudinal studies, which provide more compelling evidence of cause–effect relations, have shown in (pre-)school aged boys that a less optimal family environment predicted higher ADHD levels [14]. In addition, in 4-year-old boys, a negative home atmosphere was significantly associated with ADHD symptoms 2.5 years later [15], but it is unknown whether this association holds for adolescents. Furthermore, little is known about the role of a positive environment in the course of ADHD symptoms, despite evidence that individuals living in a supporting environment generally tend to have better developmental outcomes across adolescence [16, 17]. One longitudinal study found that higher levels of parental involvement predicted reduced symptoms of ADHD symptoms in young children [18]. In school age boys, a more optimal family environment predicted lower ADHD symptoms [19, 20]. In recent years, there has been more attention for (prospective) associations between ADHD symptoms and family functioning, but those studies often focused on parental ADHD symptoms rather than the broader family environment (e.g., [21, 22]). Prospective associations between adolescents’ ADHD symptoms and the socio-emotional quality of family functioning, which we here refer to as the family climate, are an understudied topic.

The school environment another important environmental factor that may be related to ADHD symptoms over time, has received little research attention, despite the fact that adolescents spent substantial amounts of time at school. In general, it is well known that classroom quality is influential in reducing ADHD symptoms by behavioral school interventions [23], but it is also possible that a negative classroom environment might exacerbating adolescents’ risk for elevated ADHD symptoms. It has frequently been reported that individuals with ADHD are more often rejected [24] or bullied by their peers than typically developing adolescents [25, 26], which may have negative effects on development. Conversely, friendships may play an important protective role against negative psychosocial outcomes for individuals with ADHD [27]. Apart from peers in the classroom, teachers make a crucial contribution to adolescents’ academic and social-emotional outcomes [28, 29], and this may well hold for ADHD symptoms as well. However, students with ADHD exhibit a variety of behaviors in the classroom that may disrupt teaching, increase teacher’s experience of stress, and may stand in the way of a supporting school climate [30]. Taken together, these studies suggest that the school climate, here defined as the extent of experienced security and comfort at school by the adolescent, may also affect the ADHD symptom course across adolescence.

Individuals differ in the extent to which they are influenced by the environment. The differential susceptibility theory states that some individuals are sensitive to negative and positive environments [31, 32]. The dopamine D4 receptor gene (*DRD4*) is proposed as one of the genetic susceptibility variants [33]. In the present study, we investigated whether this gene functions as a moderator of the effect of the family and school climate on ADHD symptoms across adolescent development. Gene–environment interaction (G × E) studies have shown that individuals carrying the *DRD4* 7-repeat are more vulnerable to negative environments and may also benefit more from supportive environments [34, 35]. In relation to the family environment, young children with the *DRD4* 7-repeat allele have been shown to be more sensitive to both positive and negative aspects of parenting [33, 35]. Another study, focusing on negative family influences, showed that children’s *DRD4* variants moderated the association between parental inconsistent discipline and the children’s ADHD [36]. In relation to the school environment, genetic moderation studies with the *DRD4* genotype have examined the influence of peers and teachers in promoting positive development. For example, children with the *DRD4* 7-repeat allele who experienced little to no peer victimization had lower levels of externalizing behaviors compared to when they experienced high amounts of peer victimization [37]. However, in a previous TRacking Adolescents’ Individual Lives Survey (TRAILS) study, in relation to delinquency, *DRD4* 7-repeat allele carriers were less sensitive to the effects of both peer victimization and social well-being [38]. A similar effect has been found for the association between teacher–student dissatisfaction and rule-breaking behaviors which was stronger for adolescents without the 7-repeat *DRD4* non-long carriers [39]. Together these findings suggest that the prospective associations between family and school climate and ADHD studied here may be moderated by *DRD4* genotype.

Within the framework of differential susceptibility theory, it is also relevant to investigate another plasticity gene, the *5*-*HTTLPR* genotype [34]. Individuals who are S-allele carriers were found to be more vulnerable to negative environments, but also profited more from a positive environment compared to L-allele homozygotes [40]. One cross-sectional study in youth aged 6–17 years showed that family conflict predicted increased inattention symptoms, whereas family cohesion predicted decreased inattention symptoms, but only for adolescents homozygous for the S-allele [41]. Furthermore, only in S-allele carriers, caregiver-reported peer problems at age 4 predicted ADHD symptoms two years later [42]. This literature thus suggests that the *5*-*HTTLPR* genotype might moderate associations between ADHD symptoms and both the family and school climate.

The present study investigated whether longitudinal bidirectional associations between the family and school climate, and ADHD symptoms across adolescent development (mean ages 11, 13.5, and 16 years) is moderated by the *DRD4* and/or *5*-*HTTLPR*. Using the Random Intercept Cross-Lagged Path Model (RI-CLPM), we aimed to distinguish between-person differences (i.e., between stable trait levels) from within-person (causal) processes over time (i.e., in change over time [43, 44]). By partialling out between- and within-person variance, more adequate inferences can be drawn regarding within-person (causal) processes in development [44], compared to conventional cross-lagged path models [45, 43]. That is, on one hand, genetic moderation may take place on the stable associations of ADHD symptoms with both family and school climate capturing the entire period of adolescence between ages 11 and 16 (i.e., moderation at the between-person level). On the other hand, genetic moderation may take place at the within-person level, where the application of the RI-CLPM allows for determining genetic moderation is present in within causal-person processes of ADHD influencing the family and school climate or vice versa during adolescence. The latter captures the dynamic interplay between genes and environment on developmental change over the life course [46], which we examined here during adolescence.

In line with most G × E research, we expected that *DRD4* 7-repeat carriers and *5*-*HTTLPR* S-allele homozygotes would be more sensitive to the effects of a more favorable family and school climate as indicated by a reduction of ADHD symptoms. Vice versa, we expected also that adolescents with these genetic variants would have higher ADHD symptoms across adolescence in less favorable family and school climates. However, the previous G × E research has been mainly cross section in nature (e.g., [33, 35, 37, 39]) and, furthermore, has not focused on within person change dynamics. Using the RI-CLPM model, we expected G × E effects on both the between-person level and the within-person level.

## Methods

### Sample

The 1848 participants were from the Tracking Adolescents’ Individual Lives Survey (TRAILS) who took part in the first (*T*1), second (*T*2), and/or (*T*3) measurement waves. TRAILS is an ongoing prospective study of Dutch adolescents with the aim to chart and explain the development of mental health from early adolescence into adulthood. The current paper concerns longitudinal data derived from two cohorts, a population-based cohort and a clinic-referred cohort. The population-based cohort comprised young adolescents from five municipalities in the north of The Netherlands, including both urban and rural areas. The inclusion of the clinic-referred cohort, which started 2 years later, was based on referral to the Groningen university child and adolescent psychiatric outpatient clinic, which has a catchment area corresponding with the recruitment areas of the population sample. About 20.8% had been referred at age ≤ 5 years, 66.1% between age 6 and 9 years, and 13.1% between age 10 and 12 years. The child’s parents or legal guardian and adolescents (≥ 12 years) provided both written informed consent prior to each wave, whereas younger participants provided verbal assent. The TRAILS study was approved by the Central Committee on Research Involving Human Subjects (Dutch CCMO). The sampling procedures, descriptive statistics, and response rates of both cohorts are well-documented in papers by De Winter et al. [47] and Oldehinkel et al. [48].

At baseline (T1), 2773 adolescents participated in the population-based (*n *= 2230) and clinic-referred cohort (*n *= 543), with response rates for both cohorts over 80% for follow-up assessment at T2 and T3. The *DRD4* and *5*-*HTTLPR* genotypes were determined for 1873 and 1788 of the 1922 adolescents who had donated DNA. Of those participants, 21 individuals with no data on any ADHD symptoms measurement were excluded. Furthermore, four adolescents had no data on either family or school climate resulting in a final sample size of 1860 participants. Of these participants, 1848 and 1763 adolescents had genetic data on the *DRD4* and *5*-*HTTLPR*, respectively.

At T1, 148 (10.7%) adolescents from the general population subsample had clinical levels of ADHD based on cut-off values from the Achenbach System of Empirically Based Assessment (ASEBA [49]). In the clinic-referred subsample, 225 (53.1%) adolescents had a life-time diagnosis of ADHD, based on the Diagnostic Interview Schedule for Children (DISC-IV parent version [50]).

### Measures

*ADHD symptoms* At all three waves, we used the seven item DSM-IV-Oriented subscale Attention-Deficit/Hyperactivity Problems of the Child Behavioral Checklist [51, 52] as a measure of ADHD symptoms. Items were scored by parents on a 3-point Likert-scale ranging from 0 (‘not true’) to 2 (‘very true or often true’).

*Family climate* Family climate was measured at T1, T2, and T3 by the General Functioning scale of the McMaster Family Assessment Device (FAD [53]). This parent-reported scale is used to assess family functioning, including statements about family communication and support (rating on a 4-points Likert scale (‘strongly disagree’–‘strongly agree’; 12 items *α* ≥ 0.85 at all times for both cohorts). Example items are ‘In times of crisis we can turn to each other for support’ and ‘Individuals are accepted for what they are’. A low score on the scale indicates a healthy family climate; a high score represents a dysfunctional family climate.

*School climate* To measure the adolescent’s experienced school climate, we used items from two scales of the Social Production Functions (SPF; see [54, 55]). The selected items from the two scales measured social support from the teacher and classmates reflecting affection and behavioral confirmation (rating on a 5-points Likert scale (‘never’–‘always’). The child rated social support from teachers originally consisted of 11 (T1 and T2) or 12 items (T3). Whereas at T1 and T2, the scale in measuring the experienced social support from classmates was a combination of friends and classmates (originally 17 items), at T3 questions only referred to classmates (originally 11 items). On this last scale we excluded the items about friends at T1 and T2. To minimize a positively biased school climate rating especially in adolescents with ADHD (i.e., positive illusory bias [56]), we focused on the most concrete items and excluded subjective items of both scales (e.g., ‘most classmates like to do things with me’; ‘most teachers like me’). Example items that were kept are ‘My teacher/most teachers I can really trust’ and ‘Most of my classmates help me when there is a problem’. Next, we created a mean score of seven items reflecting school climate and scores were then transformed such that, in line with the family climate rating, a low score indicates a healthy school climate and a high score a dysfunctional school climate. Internal consistency of the seven item school climate rating was acceptable to good (Cronbach’s *α* at T1: .81; at T2: .76, and at T3: .76).

*Genotyping* DNA was extracted from blood samples (*n* = 1525) or buccal swabs with a Cytobrush (*n* = 335) using a manual salting out procedure as described by Miller et al. [57] and was collected at T2 for the clinic-referred cohort and at T3 for the population-based cohort. Genotyping of the length polymorphisms *DRD4* was done at the Research lab for Multifactorial Diseases within the Human Genetics department of the Radboud University Nijmegen Medical Centre in Nijmegen, The Netherlands. The 48 bp direct repeat polymorphism in exon 3 of *DRD4* was genotyped on the Illumina BeadStation 500 platform (Illumina.). Three percent blanks and duplicates between plates were taken along as quality controls during genotyping. Determination of the length of the alleles was performed by direct analysis on an automated capillary sequencer (ABI3730, Applied Biosystems, Nieuwerkerk a/d IJssel, The Netherlands) using standard conditions. Call rate for *DRD4* was 99.4%.

Genotyping of the length polymorphisms *5*-*HTTLPR* by simple sequence length analysis (call rate 91.6%) and the SNP rs25331 (A/G SNP in *L 5*-*HTTLPR*) by a custom-made TaqMan assay (Applied Biosystems; call rate 96.5%) was also done at the Research lab for Multifactorial Diseases within the Human Genetics department of the Radboud University Nijmegen Medical Centre in Nijmegen, The Netherlands. Concordance between DNA replicates showed an accuracy of 100%. All *lg* alleles were recoded into S, because it has been shown that this polymorphism represents low serotonin expression comparable to the S allele [58], while *la* was recoded as L. Based on these alleles, we refer to the functionality of the expressed transporter as low (SS), intermediate (LS), and high (LL) expression. Given previous G × E research examining (un)supportive environments of individuals [31, 34], we considered the 7-repeat allele of the *DRD4* gene and the SS-allele of the *5*-*HTTLRPR* gene as the ‘plasticity’ alleles.

*ADHD medication use* Methylphenidate, dexamphetamine, and atomoxetine were coded as 0 = no use or 1 = use of any of these three, at any time in the preceding year at T1, T2, or T3. This variable served as a covariate. The RI-CLPM corrects for stable covariates (e.g., sex, socio-economic status) in the random intercepts.

### Data analyses

Using M*plus* [59] two RI-CLPM [43, 44] multigroup analyses were fitted to the data to examine longitudinal associations from T1 to T2 (2 year-follow-up) and from T2 to T3 (3 year follow-up) between ADHD symptoms, family climate, and school climate, for *DRD4* and *5*-*HTTLPR* genotypes, separately. Model fit was evaluated based on the Chi square (*χ*^2^) goodness-of-fit test, the Comparative Fit Index (CFI), and the root-mean-square of approximation (RMSEA). Values for the CFI should preferably be larger than .95 [60], and RMSEA should be below .08, and preferably below .05 [61]. For nested model comparisons we used Δ*χ*^2^ difference tests [62]. Missing data were handled using the full information maximum likelihood (FIML) method.

**Fig. 1:**
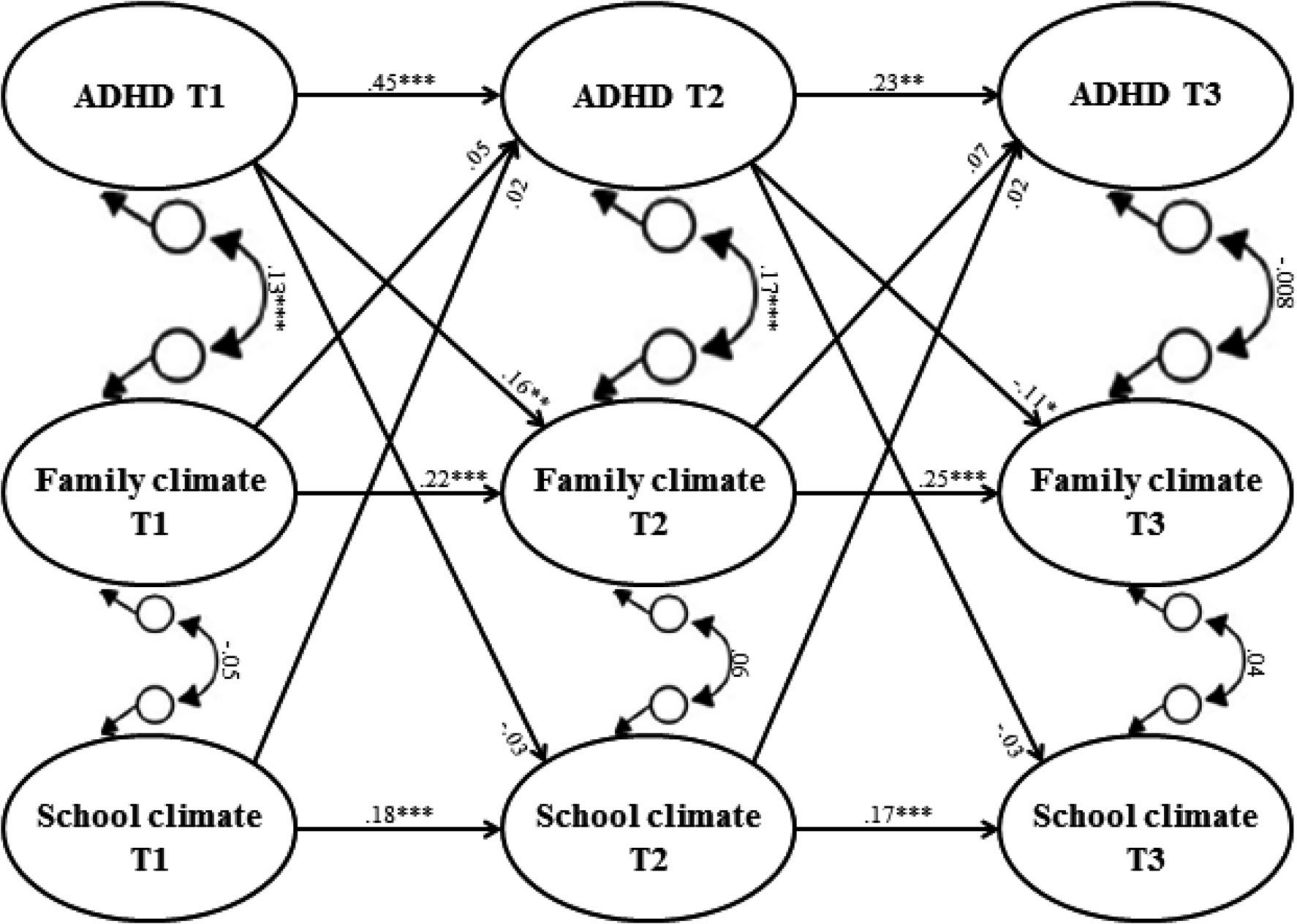
Standardized path coefficients of within-person level from the random intercept cross-lagged panel model for the associations between ADHD symptoms and family and school climate across adolescence while controlling for ADHD medication at all time points. *ADHD* attention-deficit/hyperactivity disorder symptoms. *p < .05, **p < .01, and ***p < .001 indicate significant path coefficients

Before model fitting, we calculated intra-class correlations (ICC; as is common in multi-level modeling) to examine the extent to which there was variance at the between-person and within-person level. Figure 1 represents the RI-CLPM as fitted to our data (for further descriptions of the RI-CLPM model, see [43, 44], which was modelled for *DRD4* and *5*-*HTTLPR* separately. In the RI-CLPM the between-person stable variance is modelled separately from within-person fluctuations over time. In this way, findings from the group level (i.e., between-person level) can, therefore, not mistakenly be interpreted as causes and effects on the individual (i.e., within-person level) level. To this end, the observed scores of our three main constructs at the three time points (i.e., ADHD symptoms, family, and school climate) and of our control variable (i.e., ADHD medication) were regressed on their own latent factors with the loadings constrained at one. The variances of the observed variables were constrained at zero, to capture all variance of the observed measures by both between-person and within-person latent variables. Next, four latent random intercepts were specified (i.e., for ADHD symptoms, family climate, school climate, and ADHD medication, separately) by constraining factor loadings at one. These latent random intercepts, which represent stable (i.e., stable trait level) between-person differences, were allowed to correlate. With this between-person stable variance separated out, the remaining variance represents the within-person variation (i.e., fluctuations) over time enabling us to understand possible (causal) processes between ADHD symptoms and both family and school climate and possibly the covariate ADHD medication use. within-person deviations are based on the differences of a persons’ expected score (i.e., the individual’s position in the population taking into account normative development) and the actual score on a construct. On the one hand, these within-person fluctuations are modelled by autoregressive paths (i.e., extent to which within-person deviations from the expected score can be predicted by within-person deviations from the expected score at a former time point; this reflects carry-over effects, which can both be negative and positive). On the other hand, within-person fluctuations are modeled by cross-lagged paths [i.e., extent to which within-person fluctuations (e.g., family climate) are predicted by within-person fluctuations in another construct (e.g., ADHD) at the previous time-point]. Furthermore, within-person correlations at time 1 between the four different constructs were modelled, representing the extent to which within-person deviations from the expected score in one construct at time 1 was associated with the within-person deviations from the expected score of the other three constructs at time 1. Finally, correlated residuals at time 2 and time 3 (i.e., correlated dynamic errors), model the extent to which within-person fluctuations in one construct are associated with within-person fluctuations in one of the other three constructs at the same time point (see for further explanation [43, 44]. The online supplement contains further details about subsequent model fitting.

## Results

### Descriptive statistics

The characteristics of the study sample are shown in Table 1, descriptives of the allelic variation of the *DRD4* and *5*-*HTTLPR* genotype are displayed in Supplementary Table 1. Intercorrelations among ADHD symptoms across the three time points were high (*r* values ranging from .69 to .81, all *p* values < .001), as were correlations among family climate (*r* values ranging from .43 to .59, all *p* values < .001), but they were small-to-moderate for school climate (*r* values ranging from .20 to .43, all *p* values < .001), for all variants of the *DRD4* and *5*-*HTTLPR* genotype. Of note, the correlations between ADHD symptoms and school climate at all time points were small (*r* values ranging from − .006 to .15, *p* values between < .001 and .90), whereas ADHD symptoms and family climate were more strongly correlated (*r* values ranging from .19 to .37, all *p* values < .001). There were no significant gene–environment correlations (*p*-values > .05).

**Table 1:** Sample characteristics of the study sample (*n *= 1860)

	Total sample	T1	T2	T3
Descriptives				
Population-based cohort (%)	1436 (77.2%)			
Male gender (%)	966 (51.9%)			
Age in years, *M* (*SD*)		11.09 (.55)	13.62 (.62)	16.16 (.69)
ADHD medication use^a^, *N* (%)		198 (10.6%)	224 (12.0%)	172 (9.2%)
Main variables				
ADHD symptoms^b^, M (SD)		0.68 (.53)	0.54 (.50)	0.48 (.46)
Family climate^c^, M (SD)		1.80 (.38)	1.68 (.41)	1.67 (.41)
School climate^d^, M (SD)		2.28 (.70)	2.38 (.61)	2.50 (.57)

*ADHD* attention-deficit/hyperactivity disorder

^a^Methylphenidate, dexamphetamine and atomoxetine use at any time during the past year

^b^Mean of 7-items DSM-IV-oriented ADHD subscale of the CBCL (Achenbach [51]; score range 0–2)

^c^Mean of 12-item Family Functioning scale of the FAD (Epstein et al. [53]; score range 0–4)

^d^Mean of seven selected items of the teacher and classmates subscales of the Social Production Functions (SPF [54, 55]; score range 0–5)

### RI-CLPM

The ICC for ADHD symptoms was .737 indicating that 73.7% of the variance in the three measures of ADHD symptoms over time is explained by differences between persons (i.e., stable trait level) over time. The remaining variance of 26.3% is explained by fluctuations within a person (i.e., in change over time). The ICCs for family climate, school climate, and covariate ADHD medication were .508, .285, and .690, respectively.

*DRD4 genotype* After it was demonstrated that there were no relations between family and school climate [Δ*χ*^2^(8) = 11.64, *p* = .17; fit unconstrained model *χ*^2^(12) = 9.70, CFI = 1.000, RMSEA = .000 (.000–.029)], we constrained these relations to zero, and subsequently performed model selection of which the first step was aimed at model simplification (Table 2: step 1.a to 1.c). Constraining the association between ADHD medication and family climate at the between-person level yielded the most substantial misfit in the model; these associations were, therefore, freely estimated. Longitudinal invariance was examined in step 2. In the first part of step 2 (step 2.1.a and 2.1.c), we found that the within-person stability paths of ADHD medication were not equal across time. In the second part of step 2 (step 2.2.1.a to 2.2.3.c), we separately tested whether the within-person stability paths of ADHD symptoms, correlated change between ADHD symptoms and family climate, and cross-lagged paths of ADHD symptoms predicting family climate were different over time. Only when Δ*χ*^2^ was not significant, were the corresponding paths considered equal across time and thereafter constrained to as such. In the third step, not included in Table 2, we found no differences for individuals with and without the 7-repeat of the *DRD4* genotype across all study variables at both the between-person and within-person level. The results of the within-person differences of this final model across the *DRD4* differences showed good model fit [*χ*^2^(97) = 91.981, CFI = 1.000, RMSEA = .000 (.000–.016)] and are presented in Supplementary Figure 1.

**Table 2:** Model fit comparisons of selected models in model simplification for the DRD4 genotype

	Model fit indices				Model comparison test			
	*χ*^2^	*df*	CFI	RMSEA		Δ*χ*^2^	Δ*df*	*p*
Step 1.a								
Unconstrained model	21.06	20	1.000	.008 (.000–.031)				
Step 1.b								
Constrained model	74.50	52	0.996	.022 (.009–.033)	1.b vs. 1.a	53.30	32	.01
Step 1.c								
Freely estimated: BP_ADHD medication–family climate_	53.41	50	0.999	.009 (.000–.024)	1.c vs. 1.a	32.34	30	.35
Step 2.1.a								
Unconstrained model ADHD medication	53.41	50	0.999	.009 (.000–.024)				
Step 2.1.b								
Constrained model ADHD medication	241.64	58	0.968	.060 (.052–.068)	2.1.b vs. 2.1.a	204.71	8	< .001
Step 2.1.c								
Freely estimated: WP_Stability path ADHD medication_	63.91	56	0.999	.012 (.000–.025)	2.1.c vs. 2.1.a	8.98	6	.18
Step 2.2.1.a								
Unconstrained model	63.61	56	0.999	.012 (.000–.025)				
Step 2.2.1.b								
Constrained model	97.31	62	0.994	.026 (.015–.035)	2.2.1.b vs. 2.2.1.a	33.70	6	< .001
Step 2.2.1.c								
Freely estimated: WP_Stability path ADHD symptoms_	64.89	60	0.999	.010 (.000–.023)	2.2.1.c vs. 2.2.1.a	1.24	4	.87
Step 2.2.2.a								
Unconstrained model	64.89	60	0.999	.010 (.000–.023)				
Step 2.2.2.b								
Constrained model	79.77	66	0.998	.015 (.000–.026)	2.2.2.b vs. 2.2.2.a	16.54	6	.01
Step 2.2.2.c								
Freely estimated: WP_Correlated change ADHD symptoms–family climate_	67.34	76	0.999	.008 (.000–.022)	2.2.2.c vs. 2.2.2.a	2.33	4	.68
Step 2.2.3.a								
Unconstrained model	67.34	76	0.999	.008 (.000–.022)				
Step 2.2.3.b								
Constrained model	98.10	72	0.996	.020 (.008–.030)	2.2.3.b vs. 2.2.3.a	35.50	8	< .001
Step 2.2.3.c								
Freely estimated: WP_Cross-lagged path ADHD symptoms–family climate_	72.12	70	1.000	.006 (.000–.021)	2.2.3.c vs. 2.2.3.a	4.73	6	.58

*CFI* Comparative Fit Index, *RMSEA* root mean squared error of approximation, *ADHD* attention-deficit/hyperactivity disorder, *BP* between-person level, *WP* within-person level

*5-HTTLPR genotype* In analyzing *5*-*HTTLPR* genotype differences between ADHD symptoms and both family and school climate, we applied the same procedure as when examining moderation of the *DRD4* genotype. Table 3 presents the steps of model selection and shows two important issues. First, in examining longitudinal invariance across all paths of ADHD medication (Table 3: step 2.1.b), we reached no convergence presumably caused by large misfit in the stability paths of ADHD medication. The large misfit was indicated when comparing the unconstrained model (Table 3: step 2.1.a) with a model, where only the ADHD medication stability paths over time were constrained [Δ*χ*^2^(3) = 346.84, *p* < .001]. We, therefore, freely estimated the stability paths, whereas other all ADHD medication paths were constrained over time (Table 3: step 2.1.c); this resulted in a non-significant difference with the unconstrained model. After examining longitudinal invariance, we checked for *5*-*HTTLPR* genotype differences. Compared to the *DRD4* genotype model, the stability paths of the school climate had to be freed across time. However, no moderation of the *5*-*HTTLPR* genotype on these additionally freed stability paths were found. Further inspection of these paths after constraining the model to be equal across groups showed no inferior fit [Δ*χ*^2^(1) = 0.07, *p* = .78] when the stability paths of family climate were variant instead of invariant over time. Therefore, stability path of school climate were subsequently constrained to be equal over time. Comparable with the *DRD4* genotype model, we did not find moderation of the *5*-*HTTLPR* genotype across all constructs at both the between-person and within-person level. In Supplementary Figure 2, the results of the within-person differences of this final model are presented, showing good model fit [*χ*^2^(159) = 149.826, CFI = 1.000, RMSEA = .000 (.000–.016)].

**Table 3:** Model fit comparisons of selected models in model simplification for the 5-HTTLPR genotype

	Model fit indices				Model comparison test			
	*χ*^2^	*df*	CFI	RMSEA		Δ*χ*^2^	Δ*df*	*p*
Step 1.a								
Unconstrained model	19.75	30	1.000	.000 (.000–.011)				
Step 1.b								
Constrained model	100.70	78	0.996	.022 (.004–.034)	1.b vs. 1.a	82.12	48	.002
Step 1.c								
Freely estimated: BP_ADHD medication–family climate_	74.44	75	1.000	.005 (.000–.023)	1.c vs. 1.a	54.68	45	.15
Step 2.1.a								
Unconstrained model ADHD medication	74.44	75	1.000	.005 (.000–.023)				
Step 2.1.b								
Constrained model ADHD medication	No convergence				No convergence			
Step 2.1.c								
Freely estimated: WP_Stability path ADHD medication_	78.20	84	1.000	.000 (.000–.019)	2.1.c vs. 2.1.a	5.58	9	.78
Step 2.2.1.a								
Unconstrained model	78.20	84	1.000	.000 (.000–.019)				
Step 2.2.1.b								
Constrained model	131.20	93	0.994	.026 (.015–.036)	2.2.1.b vs. 2.2.1.a	62.78	9	< .001
Step 2.2.1.c								
Freely estimated: WP_Stability path ADHD symptoms_	91.57	91	1.000	.005 (.000–.023)	2.2.1.c vs. 2.2.1.a	15.41	6	.02
Step 2.2.1.d								
Freely estimated: WP_Stability path school climate_	81.97	87	1.000	.000 (.000–.020)	2.2.1.d vs. 2.2.1.a	3.90	3	.27
Step 2.2.2.a								
Unconstrained model	81.97	87	1.000	.000 (.000–.020)				
Step 2.2.2.b								
Constrained model	101.95	96	0.999	.010 (.000–.025)	2.2.2.b vs. 2.2.2.a	22.33	9	.008
Step 2.2.2.c								
Freely estimated: WP_Correlated change ADHD symptoms–family climate_	86.93	93	1.000	.000 (.000–.019)	2.2.2.c vs. 2.2.2.a	4.87	6	.56
Step 2.2.3.a								
Unconstrained model	86.93	93	1.000	.000 (.000–.019)				
Step 2.2.3.b								
Constrained model	120.02	105	0.997	.016 (.000–.027)	2.2.3.b vs. 2.2.3.a	35.77	12	< .001
Step 2.2.3.c								
Freely estimated: WP_Cross-lagged path ADHD symptoms–family climate_	96.13	102	1.000	.000 (.000–.019)	2.2.3.c vs. 2.2.3.a	9.23	9	.42

For abbreviations see Table 2

*Overall model* Since we did not find moderation of the *DRD4* and *5*-*HTTLPR* genotypes, results on the associations between ADHD symptoms and the family and school climate are shown for the total sample. Figure 1 illustrates the within-person differences between ADHD symptoms and the family and school climate independent of genotype for all participants (*n* = 1863) with good model fit [*χ*^2^(35) = 43.87, CFI = 0.999, RMSEA = .012 (.000–.021)]. At the between-person level, there were small-to-moderate correlations between the stable traits of ADHD symptoms with family climate (*r* = .38, *p* < .001) and school climate (*r* = .23, *p* < .001). Thus, higher levels of ADHD symptoms across the three measurement waves coincide with less favorable family and school climates (and lower levels of ADHD symptoms with more favorable family and school climates). Family and school climate were positively correlated (*r* = .18, *p* < .001). Results further indicate a substantial stable association of ADHD medication with ADHD symptoms (*r* = .54, *p* < .001) and a less favorable family climate (*r* = .23, *p* = .001).

After the stable ‘trait-variance’ of ADHD symptoms, family climate, school climate, and medication use were partialled out of the model, we found small-to-large within-person changes over time. First, all within-person stability paths of school and family climate were significant and invariant over time. However, the within-person stability paths of ADHD symptoms were not equal over time, since betas were larger from T1 to T2 than from T2 to T3. This suggests that as adolescents grow older, they are approaching their expected score. Second, there was a small positive correlation between ADHD symptoms and family climate at T1. This was about half as strong as the stable between-person differences suggesting that most of the variance in the data can be accounted for by trait-like rather than state-like associations (although these associations may, of course, still be confounded by currently not studied variables). Third, the correlated changes between ADHD symptoms and family climate were differentiated between T2 and T3. The significantly correlated residuals at T2 showed that the within-person fluctuations in ADHD symptoms was associated with the within-person fluctuations in family climate. This finding indicates that when an individual’s level of ADHD symptoms increases between two adjacent measurements, the individual’s family climate becomes less favorable (or vice versa). Note that this is a correlation between dynamic errors; they are not due to the change in either study variable from 2 years earlier, nor by medication use which is part of the model, but rather by an un-modelled time-variant third factor. This association represented by the correlated change was absent at T3. Finally, the within-person cross-lagged paths of ADHD symptoms in predicting the family climate were different across time. When an individual scored higher than expected on ADHD symptoms at T1, the same individual scored lower on family climate at T2. However, the opposite pattern was found between T2 and T3, where an individual with a higher than expected score on ADHD symptoms at T2 predicted a more favorable family climate at T3. However, changes in family climate had no lagged effects on changes in ADHD symptoms over time.

## Discussion

The current study examined whether bidirectional associations between ADHD symptoms and the family and school climate were moderated by the plasticity genes *DRD4* and *5*-*HTTLPR* across adolescence (mean ages 11, 13.5, and 16 years) in a large pooled population and clinic-referred sample. We did not find genetic moderation by the *DRD4* or *5*-*HTTLPR* genotype, neither at the between-person nor the within-person level. Independent from the *DRD4* and *5*-*HTTLPR* genotype, we found important trait associations (i.e., between-person level) between ADHD and the family and school climate which indicated that adolescents with higher stable ADHD symptom levels lived in a less favorable family climate, and experienced a less favorable school climate across adolescence. Moreover, our results suggest causal effects between ADHD symptoms and the family climate (i.e., within-person level). That is, ADHD symptoms at age 11 predicted a less favorable family climate at the age of 13.5, while ADHD symptoms at age 13.5 predicted a more favorable family climate at age 16. No evidence was found for family climate altering ADHD symptoms over time, nor for change processes between ADHD and school climate at the within-person level.

Using an advanced methodological approach that separates between-person differences from within-person processes, we found that ADHD symptoms and family climate were predominantly associated at the between-person level (i.e., the stable trait) compared to the within-person level. The link between ADHD symptoms and a less favorable family climate is consistent with previous literature [11, 12, 63], but does not inform us about the within-person changes that may take place during adolescence. Based on our findings between ADHD symptoms and family climate on the within-person level (i.e., in change over time) we found some evidence on prospective change in the link between ADHD symptoms and the family climate across adolescence. The findings that relate to change yielded two important conclusions about possible causal processes between ADHD symptoms and family climate. First, we found evidence, in line with the results of the between-person level, that ADHD symptoms at age 11 prospectively predicted a lower family climate at age 13.5. On top of the stable characteristics between ADHD symptoms and family climate at the between-person level, high ADHD symptom levels predicted an even worse family climate 2.5 years later. However, the converse held later in adolescence, such that ADHD symptoms at age 13.5 predicted a more favorable family climate at age 16 years. This latter finding may at first glance seem unexpected, given that previous literature showed lower quality of family life among older youth, and high caregiver strain in families with ADHD [64]. It has to be kept in mind, however, that little is known about within person change processes, and that we also confirm this negative association between ADHD symptoms and family climate as being consistently present. There are two possible explanations for the unexpected finding which may be driven by those with or without ADHD symptoms. One explanation is that this finding might be a result of normative adolescent development, as adolescents spent less time at home [65] resulting in less parenting strain especially in families with ADHD. A second explanation again related to normative development would be that the positive effect of ADHD on family climate in the later phase of adolescence is in particular driven by the typically developing part of the sample without ADHD symptoms. That is, parents of adolescents without ADHD, at the most intense period of puberty when hormonal changes contribute to greater mood disruptions [66], may see the family climate as changing for worse. This in contrast with parents of children with ADHD who have been exposed to difficult behavior for a long time may actually during puberty not experience any surplus change for worse. Although our findings clearly need replication, and the explanations offered for our findings are currently speculative, they illustrate that, by distinguishing stable traits (i.e., between-person level) from change processes that occur between adolescents and their families (i.e., within-person level [43, 44]), more valid estimates of dynamic processes between the individual and his or her environment can be obtained. Future studies, using larger numbers of adolescents with and without ADHD are required to investigate to which extent effects (if replicated) are explained by ADHD or non ADHD individuals.

The second important finding related to processes over time is that we did not find evidence for possible causal effects of family climate on ADHD symptoms. That is, worsening of family climate did not increase ADHD symptoms or, conversely, improvement in family climate did not lead to reductions in ADHD symptoms. This contrasts with the suggestion of Johnston and Mash [67] that negative family relations might influence the continuation of ADHD symptoms, or, with findings from a meta-analysis of Coates et al. [68], showing the attenuation of ADHD following parent training intervention. But again, the existing literature has so far documented between person associations without disentangling the stable status quo from the change processes. These findings from the literature may thus be consistent with our stable findings that positive family climate co-occurs with less ADHD symptoms (or negative family environment with more ADHD symptoms). Alternatively, our null-finding may reflect the observational study design with large time lags of 2–3 years between measurements. Positive changes in the family climate may be more likely and, therefore, more easily detected, following treatment interventions A second alternative explanation is that the parenting environment, which is part of the family climate, is more critical in childhood than in adolescence [69]. It is, therefore, possible that the family climate influences ADHD symptoms especially at younger ages than examined here. Overall, our findings should be replicated.

Our finding of higher stable ADHD symptoms correlated with a less positive school climate across adolescence at the between-person level is in line with previous studies documenting an association between a good school climate and fewer student- and teacher reported internalizing and externalizing problems [70, 71, 72 in press]. However, there were no directional effects on the within-person level between ADHD symptom levels and the school climate in our study. While this may indicate that the two are not causally linked across adolescence, an alternative explanation for not finding these cross-lagged dynamical effects in the current study may lie in the large time lag, which might not capture reciprocal person- environment change. That is, it is to be expected that, compared to the family environment, there is a multitude of changes during two measurement waves, including changing peers and teachers. In light of these constantly changing circumstances in the adolescent’s life outside the family environment, the absence of effects may indicate that the school climate is too distal and has changed too much to be of influence 2–3 years later. Vice versa, adolescence is a phase of considerable change in ADHD symptoms. The impulsive ADHD symptoms at for example age 11 may have receded at age 13.5 and may play no role in the renewed school environment at age 13.5. We recommend that causal effects of a positive school environment on ADHD symptoms be studied at smaller time intervals.

In contrast to previous studies (e.g., [33, 34]), adolescents with the *DRD4* 7-repeat or being homozygous for the S-allele of the *5*-*HTTLPR* were not more responsive to the family or school environment in a ‘for better and worse’ manner. Thus, our study found no support for the differential susceptibility hypothesis. Previous studies on differential susceptibility have focused mostly on differences between individuals rather than the processes of change as they take place within the lives of an individual (i.e., within-person level), and have not applied the RI-CLPM model which separates these two levels. Thus, in so far as our data would show support for differential susceptibility, based on the literature we did expect this to become apparent at the between- subject level, while potential effects at the within-subject level would be novel. We had hoped that our developmental perspective would elucidate conflicting results especially about the ‘risk’ variant of the *DRD4* genotype [37–39]. The absence of G × E effects could be due to the lack of power despite a relatively large sample size of almost 2000 participants. It could also be argued that the absence of G × E associations between ADHD symptoms and both family and school climate indicates these associations do not exist. There is a strong publication bias towards positive findings in the G × E literature [73], which might result in the absence of comparable (i.e., null-finding) published G × E studies.

### Strengths and limitations

A strength of this study was the use of a large, longitudinal dataset and the application of an advanced methodological approach that separates between-person (i.e., stable trait levels) and within-person (i.e., causal processes) associations, providing clear evidence for links between ADHD symptoms and both family and school climate independent from the *DRD4* and *5*-*HTTLPR* genotypes across adolescent development. Some limitations should be taken into account when interpreting the current findings. First, assessment of the family climate as reported by parents does not necessarily inform us how adolescents themselves experienced the family climate. Another disadvantage of relying on parent reports of the family climate is that method variance may in part explain the associations with ADHD symptom levels (as these were also rated by the parents). However, it has been found that parent-reports of the home environment are well in line with child-reports [74]. Nevertheless, future research could benefit from self-ratings of both the family and school climate and the assessment of ADHD symptoms by at least two independent evaluators in different contexts. Second, the rating of the school climate was a compound measure of items relating to peers and teachers. This may have concealed parts of the effects (peers and teachers may not be equally important to adolescents). We also cannot be certain about the extent in which Positive Illusory Bias may have been involved in our school climate rating, despite our selection of the least subjective items. Third, adolescents are embedded in multiple other contexts than the family and school (e.g., sports team) that might affect ADHD symptoms across adolescence and which need to be included in future research. Fourth, while we controlled for ADHD medication, for a better understanding of causal processes at the within-person level other factors that are subjective to change (e.g., time spent outside home) should be incorporated as covariates in future investigations. Finally, it should be noted that present findings on the full spectrum of ADHD symptoms in adolescents may not generalize to clinical samples selected for being diagnosed with ADHD, or to other age groups.

## Conclusions

Our study provides support that adolescents with ADHD symptoms and a less favorable family and school climate are tightly interwoven, as shown by stable between-person differences across adolescence. Moreover, within-person changes over time point to a causal role of ADHD symptoms on the family climate. These effects were independent from both the *DRD4* and *5*-*HTTLPR* genotype; thus, no evidence for differential susceptibility to the environment was found. To the extent that our findings on change processes may seem, unexpected, it is important to repeat that the existing knowledge base is confounded by stable associations between ADHD symptoms and family climate which have not been partialled out in previous studies. We recommend replication of our study applying the RI-CLPM to tease stable associations and change processes apart.

## Electronic supplementary material

Below is the link to the electronic supplementary material.

Supplementary material 1 (DOCX 228 kb)
