# Supplementary material for: ADHD symptoms across adolescence: the role of the family and school climate and the DRD4 and 5-HTTLPR genotype
Source: Eur Child Adolesc Psychiatry. 2019 Oct 18;29(8):1049–61. doi: 10.1007/s00787-019-01424-3 (PMC7369263; doi:10.1007/s00787-019-01424-3)
Supplement: Supplementary file 1 — Supplementary material 1 (DOCX 228 kb) [file 787_2019_1424_MOESM1_ESM.docx]

**Supplementary information**

Once this baseline RI-CLPM model was specified, we aimed for model simplification. We constrained non-theoretical paths to zero (i.e., cross-lagged paths between family and school climate, and both *between-person* and *within-person* associations between ADHD medication and family and school climate) and checked if this deteriorated model would fit. using nested model comparison with the Δχ^2^ difference test. If model fit deteriorated significantly which was indicated by a significant Δχ^2^ difference test, we checked which individual parameter was causing the most substantial misfit in the model. This parameter was then freely estimated and we tested the adjusted model to the constrained model, for as long as these models differed significantly. Step 1 thus led to the most simple baseline model with paths constrained to zero according to our paths of interest whenever the data indeed confirmed this.

Second, in order to model possible change over time, we examined longitudinal invariance to determine whether repetitive parameters (i.e., carry over stability paths, correlated change, and cross-lagged paths) differed across time. With equality constraints at corresponding parameters over time (i.e., stability paths, correlated change, and cross-lagged paths) we compared a range of nested models using Δχ^2^ difference tests (Satorra & Bentler, 2001). If significant, we allowed parameter estimates to differ over time (e.g., if model fit indicated that the association between school climate and ADHD from T1 to T2 would be different from that from T2 to T3). Of note, longitudinal invariance was first tested for ADHD medication and thereafter for the three main constructs of interest, (i.e., ADHD symptoms, and both family and school climate) for stability paths, correlated change, and cross-lagged paths, once at a time. All non-repetitive paths were freely estimated (i.e., *within-person* correlations at T1 and *between-person* differences among the four constructs). Step 2 thus led to full model estimation of all concurrent and across-time relations between ADHD, family climate, school climate, and covariate ADHD medication use.

Third, in order to examine differences across the *DRD4* genotype, nested models were compared with Δχ^2^ difference test (Satorra & Bentler, 2001). We used three sequential steps in analyzing which paths were invariant across the *DRD4*: paths involving ADHD medication, paths of little substantive interest (i.e., time-[in]variant stability paths, time-[in]variant correlated change, and *within-person* correlation T1), and paths of strong substantive interest (i.e., time-[in]variant cross-lagged paths, and *between-person* differences). When paths were invariant across the *DRD4*, we constrained them across the *DRD4* variant. Step 3 thus led to differences across the *DRD4* genotype between ADHD, family climate, school climate, and covariate ADHD medication use. All these three steps were then repeated for the *5-HTTLPR* genotype.

Supplementary Table 1.

*Sample characteristics per DRD4 (n = 1,848) and 5-HTTLPR* *(n = 1,763)* *status*

|  | |  | ***DRD4 genotype*** | |  | ***5-HTTLPR genotype*** | | | |  |
| --- | --- | --- | --- | --- | --- | --- | --- | --- | --- | --- |
|  | |  | ***7-repeat absent***  *n* = 1,167 (63.1%) | ***7-repeat present***  *n* = 681 (36.9%) | ***Test statistic*** | ***SS***  *n* = 452 (25.6%) | ***LS***  *n* = 871 (49.4%) | | ***LL***  *n* = 440 (25.0%) | ***Test statistic*** |
| Population-based cohort (%) | |  | 890 (76.3%) | 536 (78.7%) | χ²(1) = 1.46 | 338 (24.3%) | 696 (50.1%) | | 355 (25.6%) | χ²(2) = 5.95 |
| Age in years, *M* (*SD*) | | T1 | 11.09 (.53) | 11.09 (.57) | *F*(1) = 0.04 | 11.07 (.54) | 11.09 (.54) | | 11.09 (.55) | *F*(2) = 0.19 |
|  |  | T2 | 13.35 (.62) | 13.38 (.61) | *F*(1) = 0.74 | 13.34 (.63) | 13.39 (.61) | | 13.36 (.60) | *F*(2) = 0.80 |
|  |  | T3 | 16.15 (.68) | 16.17 (.69) | *F*(1) = 0.17 | 16.15 (.69) | 16.18 (.68) | | 16.15 (.70) | *F*(2) = 0.37 |
| Male gender (%) | |  | 581 (49.8%) | 375 (55.1%) | χ²(1) = 4.80* | 246 (54.4%) | 435 (50.0%) | | 221 (50.0%) | χ²(2) = 2.60 |
| ADHD medication use^a^, *N* (%) | | T1 | 134 (11.5%) | 62 (9.1%) | χ²(1) = 2.57 | 49 (10.8%) | 99 (11.3%) | | 34 (7.7%) | χ²(2) = 4.36 |
|  |  | T2 | 151 (12.9%) | 71 (10.4%) | χ²(1) = 2.57 | 51 (11.3%) | 106 (12.2%) | | 45 (10.2%) | χ²(2) = 1.11 |
|  |  | T3 | 116 (9.9%) | 55 (8.1%) | χ²(1) = 1.78 | 39 (8.6%) | 84 (9.6%) | | 31 (7.0%) | χ²(2) = 2.49 |
| *Main variables* | | | | | | | | | | |
| ADHD symptoms^b,e^, *M* *(SD*) | | T1 | 0.69 (.53) | 0.67 (.52) | *F*(1) = 0.30 | 0.73 (.54) | 0.66 (.53) | 0.66 (.49) | | *F*(2) = 2.57 |
|  |  | T2 | 0.54 (.50) | 0.54 (.48) | *F*(1) = 0.26 | 0.57 (.50) | 0.52 (.50) | 0.52 (.46) | | *F*(2) = 1.49 |
|  |  | T3 | 0.49 (.47) | 0.47 (.42) | *F*(1) = 1.13 | 0.51 (.47) | 0.48 (.46) | 0.45 (.43) | | *F*(2) = 1.69 |
| Family climate^c,d^, *M* *(SD*) | | T1 | 1.79 (.37) | 1.80 (.37) | *F*(1) = 0.15 | 1.81 (.38) | 1.80 (.38) | 1.78 (.36) | | *F*(2) = 0.75 |
|  |  | T2 | 1.68 (.41) | 1.67 (.42) | *F*(1) = 0.18 | 1.68 (.43) | 1.68 (.41) | 1.66 (.40) | | *F*(2) = 0.50 |
|  |  | T3 | 1.66 (.41) | 1.69 (.42) | *F*(1) = 1.61 | 1.70 (.42) | 1.68 (.42) | 1.62 (.39) | | *F*(2) = 3.63* |
| School climate^c,e^, *M* *(SD*) | | T1 | 2.28 (.69) | 2.28 (.70) | *F*(1) = 0.06 | 2.27 (.69) | 2.29 (.70) | 2.28 (.72) | | *F*(2) = 0.22 |
|  |  | T2 | 2.37 (.61) | 2.39 (.60) | *F*(1) = 0.49 | 2.38 (.60) | 2.38 (.59) | 2.35 (.63) | | *F*(2) = 0.31 |
|  |  | T3 | 2.50 (.57) | 2.51 (.56) | *F*(1) = 0.07 | 2.54 (.56) | 2.61 (.57) | 2.46 (.57) | | *F*(2) = 2.05 |

*Note.* *DRD4* = Dopamine D4 Receptor Gene *ADHD* *=* Attention-Deficit/Hyperactivity Disorder

* *p* < .05, ** *p* <.01, *** *p* < .001

^a^ Methylphenidate, dexamphetamine and atomoxetine use at any time during the past year (1) versus non-use (0)

^b^ Mean of 7-items DSM-IV-oriented ADHD subscale of the CBCL (Achenbach, 1991; score range 0-2)

^c^ T1-T2 < T2-T3 at *p* < .001.

^d^ Mean of 12-item Family Functioning scale of the FAD (Epstein et al., 1983; score range 0-4)

^e^ Mean of 7 selected items of the teacher and classmates subscales of the Social Production Functions (SPF; Ormel et al., 1997, Ormel, 2002; score range 0-5).

*
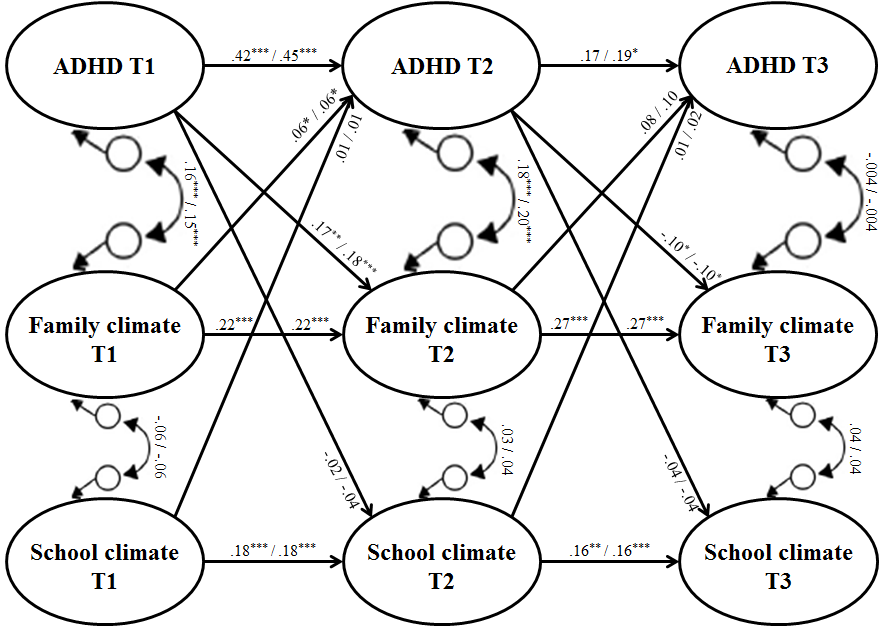
Supplementary Figure 1.* Standardized path coefficients of within-person level from the random intercept cross-lagged panel model for the associations between ADHD symptoms and family and school climate across adolescence while controlling for ADHD medication at all time points.

*Note. ADHD* = Attention-Deficit/Hyperactivity Disorder.

Left, and right path coefficients represent absences (*n* = 1,167), and presence 7-repeat variant (*n* = 681) of the *DRD4* genotype, respectively.

**p* < .05, ***p* < .01, and ****p* < .001 indicate significant path coefficients within the respective variation of the *DRD4*.

**
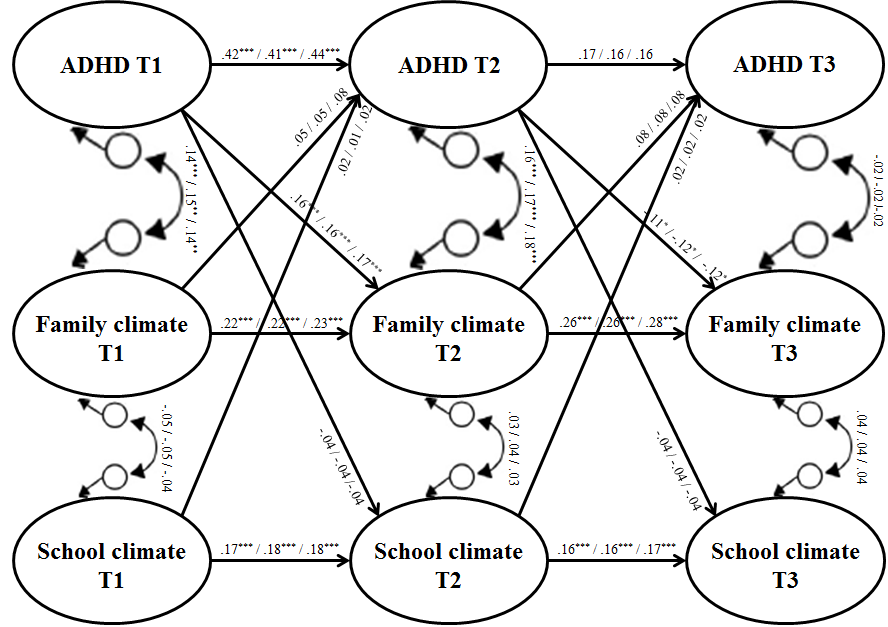
**

*Supplementary Figure 2.* Standardized path coefficients of within-person level from the random intercept cross-lagged panel model for the associations between ADHD symptoms and family and school climate across adolescence while controlling for ADHD medication at all time points.

*Note. ADHD* = Attention-Deficit/Hyperactivity Disorder.

Left, middle, and right path coefficients represent S-allele homozygotes (*n* = 452), L-allele heterozygotes (*n* = 871), and L-allele homozygotes (*n* = 440) of the *5-HTTLPR* genotype, respectively.

**p* < .05, ***p* < .01, and ****p* < .001 indicate significant path coefficients within the respective variation of the *5-HTTLPR*.
